# Improving Tribological Performance of Poly(phenylene sulfide) by Incorporating PTFE Fillers: The Influence of Filler Type and Concentrations

**DOI:** 10.3390/polym17091222

**Published:** 2025-04-29

**Authors:** Junpeng Li, Jixiang Li, Jianbo Xiang, Xiaoxi Gong, Peng Xie, Yang Chen, Mei Liang, Huawei Zou, Shengtai Zhou

**Affiliations:** 1 National Key Laboratory of Advanced Polymer Materials, Polymer Research Institute, Sichuan University, Chengdu 610065, China; 2 China Bluestar Chengrand (Chengdu) Testing Technology, Co., Ltd., Chengdu 610041, China

**Keywords:** poly(phenylene sulfide), polytetrafluoroethylene, microstructure, tribological performance

## Abstract

Poly(phenylene sulfide) (PPS) is a high-performance thermoplastic engineering material with excellent comprehensive performance that finds application in many fields due to its good processability, excellent heat resistance, and mechanical properties. However, the poor friction and wear properties of PPS limit its wide application in industrial sectors. In this work, polytetrafluoroethylene (PTFE) was adopted as the solid tribo-modifier to improve the tribological performance of PPS. The efficacy of using three types of PTFE fillers, namely PTFE fiber, micropowder, and nanopowder, was comparatively investigated. The results revealed that the incorporation of PTFE was beneficial to improving the tribological properties of PPS and PTFE nanopowders, which were prepared by irradiation treatment technology that demonstrated the best modification effect in terms of both tribological and mechanical performance among the studied systems. In addition, the coefficient of friction and specific wear rate of PPS composites with 30 wt% nanopowders reached 0.165 and 3.59 × 10^−5^ mm^3^/Nm, respectively, which were 70.7% and 99.0% lower than their pure PPS counterparts. The above finding was attributed to the improved compatibility between the PTFE nanopowders and the PPS substrate as well as the easier formation of intact PTFE transfer film on the contact surface. This work shows some perspective for designing self-lubricating polymer composites that broaden their application in industrial sectors.

## 1. Introduction

The friction-induced energy loss and part failure are prevalent in industrial sectors, which have a significant impact on the economy and environments [1]. Globally, it is estimated that about 1/3 of primary energy is consumed in friction-related scenarios, and the malfunction of structural components caused by wear may lead to catastrophic disasters in high-tech engineering sectors such as automotive, aerospace, and nuclear industries, among others [2,3]. Thus, it becomes imperative to improve the tribological properties of friction pairs to sustain the service life of engineering parts [4,5,6,7].

Polyphenylene sulfide (PPS) is a high-performance engineering thermoplastic that possesses excellent mechanical performance and good processability, and it is widely used in automotive, electronics, and aerospace industries [8,9]. However, the high coefficient of friction (COF, >0.55) and severe wear loss of pure PPS [10,11] limit its application in friction-related sectors. A series of modification methods, such as adding solid lubricants [12,13,14], using fiber/fabric reinforcements [15,16], blending with other functional components [17,18], coating and biomimetic lubrication [19], tuning the filler orientation [20], and constructing filler network [21,22], are used to improve the tribological properties of polymers. In addition, Kumr and Saha [15] studied the influence of agricultural waste (corncob filler) particle size on the mechanical and tribological performance of epoxy biocomposites. Their work indicated that there was an optimal particle size in tuning the properties, and the samples that were modified using 125–200 μm corncob filler demonstrated the best performance in terms of mechanical properties, tribological performance, and thermal stability.

It is proven that polytetrafluoroethylene (PTFE) is an effective solid lubricant that significantly improves the tribological properties of polymer composites [23,24,25,26,27]. However, it is intrinsically inert and nonpolar, which exhibits poor distribution in polymer substrates, which deteriorates the mechanical properties [28]. Shojaei et al. [29] modified PTFE with sodium naphthalene dicarboreal, and the obtained PTFE (i.e., g-PTFE) was used as a solid lubricant to improve the friction and wear properties of polyamide 66 (PA66). Their results showed that when compared with untreated PTFE, both the mechanical and tribological properties of g-PTFE-filled PA66 composites were significantly improved, which was attributed to the enhanced interaction between g-PTFE and PA66. Although the chemical treatment method improves the compatibility between PTFE and polymer substrate [30,31,32], it suffers cumbersome preparation steps and environmental pollution [33], which restricts its wide application in industrial sectors.

Unlike the chemical treatment method, radiation treatment technology is a simple, efficient, and clean method that is widely adopted to modify the surface property of fillers [34,35,36,37]. Kamga et al. studied the efficacy of using radiation-modified PTFE to improve the tribological properties of PA [38]. Their results indicated that the use of irradiated PTFE (i-PTFE) improved the tribological properties of PA more than those with untreated PTFE. In previous studies, adding i-PTFE was found effective in terms of improving the tribological properties of polyoxymethylene (POM) [39], poly(arylene ether nitrile) [40], and PPS [41]. Although many studies were devoted to studying the influence of modified PTFE on the tribological properties of thermoplastic polymers, a comparison of tribological, mechanical, and morphological properties of PPS-based composites by considering morphological effect of PTFE is still lacking. To bridge this knowledge gap, this work adopted three types of PTFE with different morphological factors as solid tribo-modifiers to improve the tribological and mechanical properties of PPS.

In the present work, a series of PPS/PTFE binary blends with different types of PTFE (i.e., PTFE fiber, micropowder, and nanopowder) were prepared by adopting the melt-processing method. The influence of the PTFE type and the concentration on the tribological and mechanical properties of PPS were studied. It was found that the mechanical properties of PPS were impaired by adding PTFE, but their tribological properties were greatly improved, especially for the PPS composites containing irradiated PTFE nanopowders. Moreover, the presence of polar groups on the surface of PTFE fillers favored their distribution in polymer substrate, which was advantageous to improving the tribological performance while still maintaining reasonable mechanical performance.

The results show that the coefficient of friction and the specific wear rate of PPS with 30 wt% irradiation-treated PTFE nanopowders reached 0.165 and 3.59 × 10^−5^ mm^3^/Nm, respectively, which were 70.7% and 99.0% lower than pure PPS. In addition, the tensile strength and flexural strength of the corresponding samples were 50.54 and 84.72 MPa, respectively. To conclude, this work provided an effective approach to prepare self-lubricating polymer composites with integrated mechanical and tribological performance that demonstrate potential application in industrial sectors.

## 2. Materials and Methods

### 2.1. Materials

PPS was purchased from Deyang Keji High-tech Material Co., Ltd. (Deyang, Sichuan Province, China). According to the manufacturer, the melt flow index of PPS is 1327 g/10 min (5 kg, 316 °C), and the melting temperature is 285 °C. The PTFE micropowder (i.e., m-PTFE, average diameter: 5 μm) and PTFE nanopowder (n-PTFE, average diameter: 100~300 nm) were purchased from Sichuan Jinhe Polymer Material Co., Ltd., (Meishan, Sichuan Province, China). According to the producer, the PTFE powders were prepared using a BFT-IV Co-60 irradiator at a dose rate of 0.75 kGy/h. Functional groups such as -COOH and -COF were present on the surface of i-PTFE, which likely improved the interaction between i-PTFE and the substrate [39,41,42,43]. PTFE fibers (f-PTFE, average length: 6 mm, average diameter: 3–5 µm) were supplied by Shandong Senrong New Materials Co., Ltd., (Zibo, Shandong Province, China). The surface of f-PTFE was free of functional groups. All materials were used as received.

### 2.2. Preparation of PPS/PTFE Composites

Prior to extrusion, raw materials were dried in a blast oven at 80 °C for 12 h. The materials were mixed in a specific weight ratio (as displayed in Table 1) and they were compounded using a parallel twin-screw extruder (TSSJ-25/33, Chengdu Tarise Chemical Engineering Co., Ltd., Chengdu, Sichuan Province, China). The temperatures from the hopper to die zones were set as follows: 230 °C, 275 °C, 285 °C, 285 °C, 290 °C, 290 °C, 290 °C, 285 °C, 285 °C, and 285 °C. The extrudates were pelletized, collected, and dried for subsequent characterizations and sample preparation.

In accordance with the requirements for tribological and mechanical tests, splines with a desired shape were injection-molded using a MA2000 servo injection molding machine (Haitian Plastics Machinery, Ningbo, Zhejiang Province, China). The temperatures from the hopper to die zones were set at 290 °C, 300 °C, 300 °C, 300 °C, and 300 °C. The injection pressure was 120 MPa and the injection speed was 75 mm/s. The formulations for the PPS/PTFE blends are presented in Table 1. Herein, the PPS samples with 5 wt% of n-PTFE were denoted as P/n-PTFE5. The same nomenclature system was applicable to the other PTFE-containing PPS blends.

### 2.3. Characterization

The tensile properties were tested as per GB/T 1040.1-2022. The size of the dumbbell-shape specimens was 150 × 10 × 4 mm^3^ and the testing speed was 10 mm/min. The flexural properties were tested according to GB/T 9341-2008. The size of specimens was 80 × 10 × 4 mm^3^ and the testing speed was 2 mm/min. Five specimens were tested for each sample to gain the average value. The measurements were conducted using a universal mechanical testing machine (Shenzhen Suns Technology Stock Co., Ltd., Shenzhen, Guangdong Province, China).

The tribological properties of PPS/PTFE blends were tested in accordance with GB/T 3960-2016 using a M-200 testing rig (Beijing Guance Instrument Co., Ltd., Beijing, China). The dimensions of specimens were 30 × 7 × 6 mm^3^. The friction ring was made by 45# steel, and the test duration was 60 min. The outer diameter (r) of the friction ring was 40 mm, and the surface roughness (Ra) was 0.7–0.9 μm. The sliding speed was 0.42 m/s, and the applied load was 200 N. Prior to measurements, the surface of the friction ring was polished using sandpaper. The average coefficient of friction (COF) was obtained by averaging the results of three replicate tests. The calculation of COF and the specific wear rate was described elsewhere [44].

The morphology of cryo-fractured surface and the worn surface of PPS-based blends were observed using a JSM-9600 scanning electron microscope (SEM, JEOL, Tokyo, Japan). Prior to observations, the fractured surface was gold-sprayed to enhance image resolution.

## 3. Results

### 3.1. Mechanical Properties

**Figure 1:**
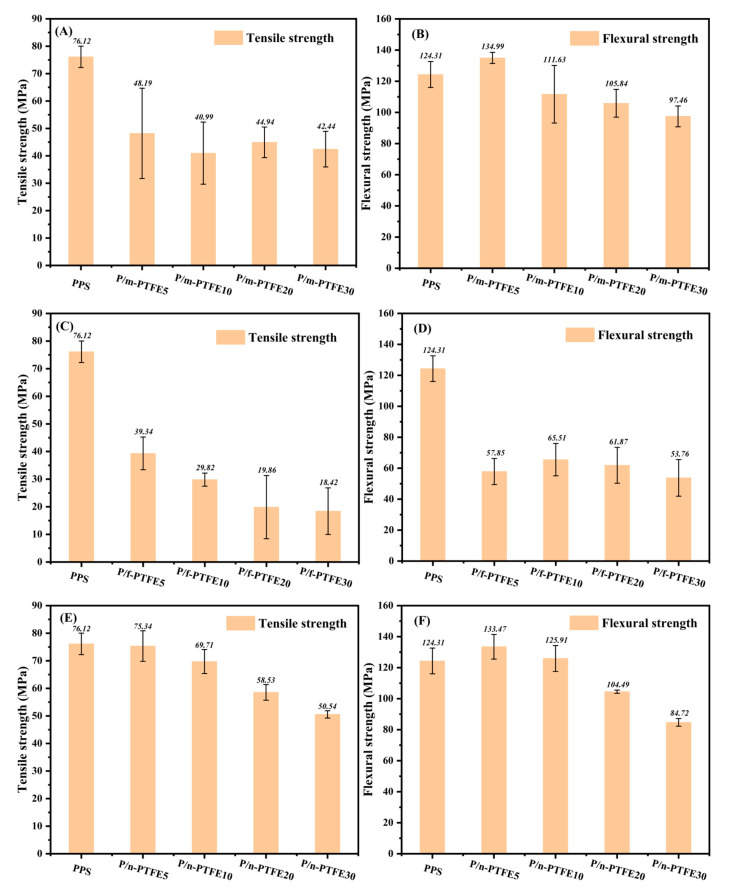
(**A**) Tensile strength and (**B**) flexural strength of P/m-PTFE blends with different filler concentrations; (**C**) tensile strength and (**D**) flexural strength of P/f-PTFE blends with different filler concentrations; (**E**) tensile strength and (**F**) flexural strength of P/n-PTFE blends with different filler concentrations. Herein, m-PTFE: PTFE micropowders; f-PTFE: PTFE fibers; n-PTFE: PTFE nanopowders.

The mechanical properties, including the tensile and flexural strength of PPS/PTFE blends, are shown in Figure 1. The tensile strength (TS) of P/m-PTFE blends showed a decreasing trend with an incremental addition of m-PTFE concentration, as displayed in Figure 1A. This was related to the formation of internal defects arising from the presence of soft PTFE particles in the host substrate, which deteriorated the TS. Figure 1B shows that the flexural strength (FS) of m-PTFE-containing blends experienced an initial increase of 5 wt%. Afterwards, a continuous decrease trend was observed with an increasing m-PTFE concentration. For example, the FS of P/m-PTFE5 reached 134.99 MPa, which was 10.68 MPa (~8.6%) higher than that of pure PPS. The reduction in FS with a further increasing m-PTFE concentration was likely attributed to the formation of defects such as filler agglomeration [45], poor interfacial interaction between m-PTFE, and PPS as well as the softness of PTFE particles [23,24,25,26].

Unlike P/m-PTFE blends, Figure 1D shows that the values of FS for P/f-PTFE blends decreased significantly with the incorporation of PTFE fibers. However, the differences in FS for P/f-PTFE blends with a different concentration of f-PTFE were not significant. In addition, the TS of P/f-PTFE decreased significantly with an increasing f-PTFE content, as shown in Figure 1C. In this scenario, it is believed that the geometry of solid lubricants played a significant role in determining the mechanical properties of the corresponding polymer blends. The incorporation of f-PTFE formed heavy entanglement, especially for high-concentration loaded blends [20], which severely impaired the overall mechanical performance.

The changing trends of TS and FS of P/n-PTFE blends are similar to those of their P/m-PTFE counterparts. To our surprise, both the FS and TS of P/n-PTFE composites remained relatively high when compared with their m-PTFE- and f-PTFE-containing counterparts. The FS of P/n-PTFE5 was higher than PPS and its TS was comparable to that of pure PPS. The above observation suggested that n-PTFE was an effective filler that had the least impact on the mechanical properties of PPS-based blends among the studied fillers. Similarly, the P/n-PTFE blends showed a lesser degree of reduction in terms of TS when compared with their P/m-PTFE and P/f-PTFE counterparts. It has been reported that oxygen-containing groups such as -OH and C=O were introduced on the surface of PTFE after the irradiation treatment [41], and the presence of functional groups improved the polarity of PTFE particles, which favored the distribution of n-PTFE in the host matrix. As a result, the relatively uniform distribution of n-PTFE and the improved interfacial interaction between n-PTFE and PPS guaranteed better mechanical performance when compared with P/m-PTFE and P/f-PTFE blends. Based on the above findings, it was concluded that adding PTFE impaired the overall mechanical properties of PPS/PTFE blends. The irradiated n-PTFE demonstrated the least influence on the mechanical properties, followed by m-PTFE and f-PTFE.

### 3.2. Friction and Wear Properties

**Figure 2:**
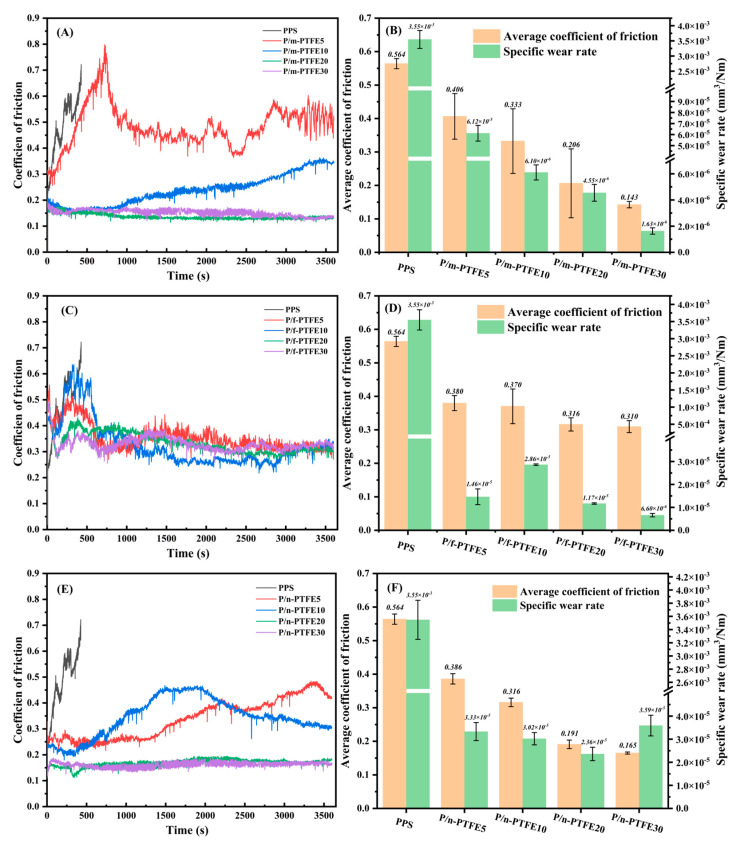
The transient COF, average COF, and specific wear rate of PTFE-containing PPS blends with different filler concentrations. (**A**,**B**) P/m-PTFE blends; (**C**,**D**) P/f-PTFE blends; (**E**,**F**) P/n-PTFE blends.

Figure 2 illustrated the trend of transient COF of PTFE-containing blends as a function of sliding time as well as the average COF and specific wear rate as a function of PTFE content. Figure 2A shows that the transient COF of pure PPS increased substantially upon the direct contact between the friction ring and sample block. As a result, friction-induced heat, which arose from the conversion of frictional work, likely induced the softening or even melting of polymer substrate, thereby resulting in a significant mass loss [46] due to the removal of wear debris. As a result, the friction test of pure PPS was terminated in the vicinity of 330 s and the wear mechanism was mainly adhesive wear.

The addition of PTFE fillers greatly improved the transient COF of PPS-based blends and the transient COF gradually became stable with increasing PTFE content, as shown in Figure 2A,C,E. Moreover, the average COF and specific wear rate of PPS-based blends decreased with the increasing PTFE concentration. Figure 2A shows that the transient COF of P/m-PTFE5 was consistently increased up to 800 s during the running-in stage. Afterwards, the transient COF of P/m-PTFE5 dropped sharply, likely due to the removal of polymer substrate that related to the softening of the sample surface. Unlike pure PPS, the tribological measurement of P/m-PTFE5 lasted for 1 h and the average COF was close to 0.4, suggesting that the wear mechanism of P/m-PTFE5 was an adhesive wear, along with the formation of transfer film, which, to some extent, mitigated the friction and wear process.

When the concentration of m-PTFE was increased to 10 wt%, the transient COF remained nearly stable during the running-in stage (from 0 to 800 s). Afterwards, there was a slight increase in transient COF with increasing sliding time. In this scenario, both the incomplete coverage of transfer film on the friction pair and the continuous build-up of frictional heat were responsible for the increase in COF. Like P/m-PTFE, the wear mechanism of P/m-PTFE10 was a partial adhesive wear. With a further increase in the m-PTFE content to 20 and 30 wt%, there was a significant improvement in tribological performance. For example, the transient COF remained nearly constant for P/m-PTFE20 and P/m-PTFE30 during the entire sliding duration (i.e., 3600 s). Under such circumstances, the formation of intact transfer film on the friction pair was indispensable for improving the tribological performance. Figure 2B shows that the average COF and specific wear rate of P/m-PTFE30 reached 0.143 and 1.63 × 10^−6^ mm^3^/Nm, respectively, which were the lowest among the studied systems.

Unlike P/m-PTFE blends, there was a gradual increase in transient COF for P/f-PTFE blends during the running-in stage (roughly before 500 s), which was related to the poorer distribution of PTFE fibers in the substrate. The subsequent decrease in transient COF was possibly attributed to the peeling-off of the matrix (especially for P/f-PTFE10), thereby leading to a sudden drop in transient COF. A similar changing trend was observed in previous studies [47,48,49]. Figure 2B shows that the average COF and specific wear rate of P/f-PTFE blends demonstrated a poor correlation with the weight fraction of fillers. The above was related to the fact that heavy filler entanglement or agglomeration likely occurred with an increasing f-PTFE content, thereby hindering the capacity to fully leverage the self-lubricating performance of PTFE. In general, both the average COF and specific wear rate of P/f-PTFE blends were inferior to those of their P/m-PTFE counterparts, indicating that m-PTFE outperformed f-PTFE in terms of reducing COF and wear loss.

The development of transient COF of P/n-PTFE blends as a function of time is presented in Figure 2E. The transient COF of P/n-PTFE5 increased significantly after being slid for about 2500 s, which was related to the accumulation of frictional heat that led to the softening of substrate. A similar increasing trend was observed for P/n-PTFE10, and the incomplete formation of transfer film and the accumulation of frictional heat were believed to be the contributing factors. When the concentration of n-PTFE was further increased to 20 and 30 wt%, the transient COF remained stable during the entire sliding duration (i.e., 3600 s). Under such circumstances, the formation of intact and continuous transfer film on the friction pair was favorable for the improvement of tribological performance. To conclude, the loading of a large amount (≥20 wt%) of m-PTFE and n-PTFE was beneficial to improving the tribological performance of PPS. Intact and continuous transfer film was easily constructed at higher PTFE concentrations, which resulted in a concurrent improvement of friction and wear properties of subsequent blends.

### 3.3. Microstructure

**Figure 3:**
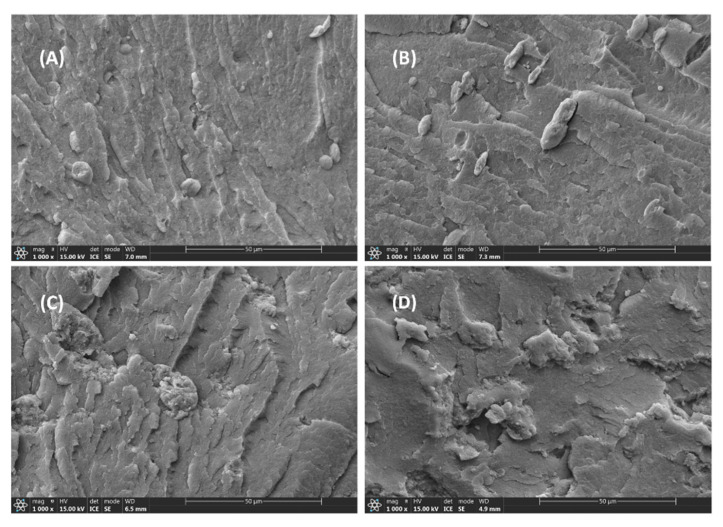
The morphology of (**A**) P/m-PTFE5, (**B**) P/m-PTFE10, (**C**) P/m-PTFE20, and (**D**) P/m-PTFE30.

The morphology of the brittle-fractured surface of P/m-PTFE blends is displayed in Figure 3. The results show that m-PTFE was relatively uniformly distributed in the host substrate, and there was a significant agglomeration of m-PTFE particles at higher filler concentrations, as displayed in Figure 3C,D. The agglomeration of m-PTFE thus greatly impaired the mechanical properties of PPS-based composites, as shown in Figure 1A,B.

**Figure 4:**
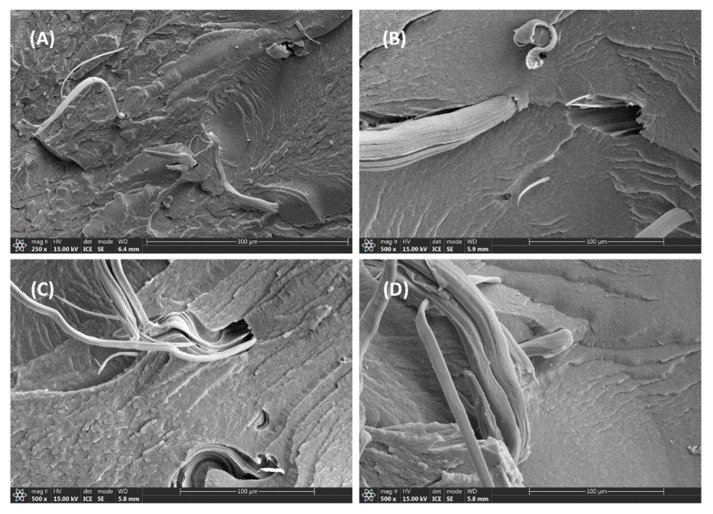
The morphology of (**A**) P/f-PTFE5, (**B**) P/f-PTFE10, (**C**) P/f-PTFE20, and (**D**) P/f-PTFE30.

The microstructure of P/f-PTFE blends as a function of filler concentration is shown in Figure 4. The results show that f-PTFE was clearly observed in the fractured surface of samples. In addition, the voids (formed by the peeling-off of f-PTFE) and obvious boundaries between f-PTFE and PPS observed in Figure 4B,C suggest poor interfacial interaction between the fillers and the host substrate. Figure 4D reveals that f-PTFE formed physical entanglements, which was unfavorable for the improvement of mechanical and tribological properties. A similar finding was reported by other research groups [16,50].

**Figure 5:**
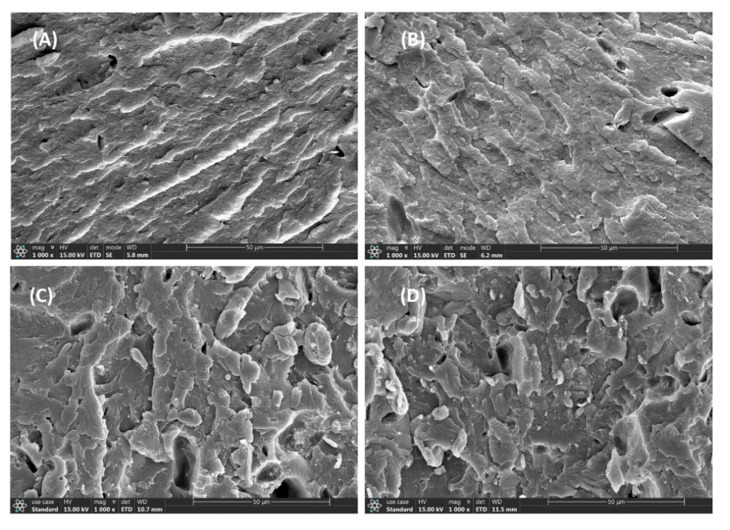
The morphology of (**A**) P/n-PTFE5, (**B**) P/n-PTFE10, (**C**) P/n-PTFE20, and (**D**) P/n-PTFE30.

The morphology of P/n-PTFE blends as a function of filler content is shown in Figure 5. The results show that n-PTFE demonstrated better distribution in PPS when compared with their m-PTFE and f-PTFE loaded counterparts. Moreover, no significant agglomeration of n-PTFE particles was observed in P/n-PTFE20 and P/n-PTFE30, as displayed in Figure 5C,D, respectively. In addition, some voids were detected in the fractured surface of P/n-PTFE blends (especially for P/n-PTFE20 and P/n-PTFE30), which was related to the removal of PTFE particles arising from the mechanical fracture behavior. Interestingly, the fractured surface of P/n-PTFE blends was rougher when compared with that of their P/f-PTFE and P/m-PTFE counterparts, which indicated that the existence of functional polar groups on the surface of n-PTFE enhanced the compatibility between n-PTFE and PPS, thereby leading to a better filler distribution and improving the mechanical and tribological performance of P/n-PTFE blends.

### 3.4. Morphology of Worn Surface

**Figure 6:**
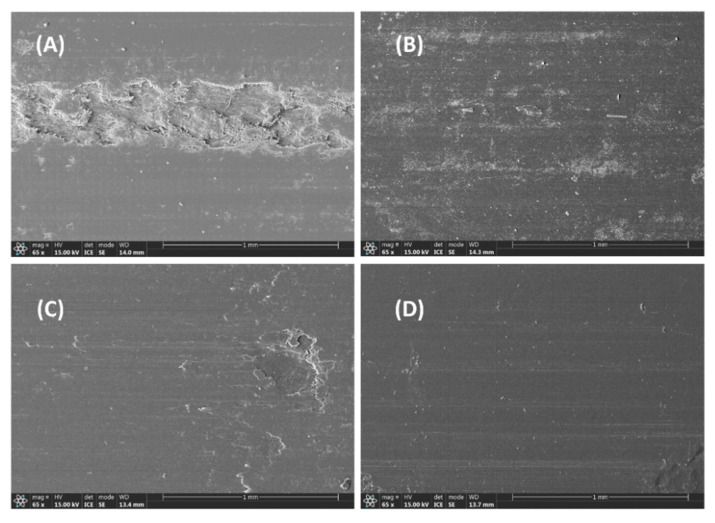
Morphology of the worn surface: (**A**) P/m-PTFE5, (**B**) P/m-PTFE10, (**C**) P/m-PTFE20, and (**D**) P/m-PTFE30.

Observing the worn surface was crucial to analyzing the friction and wear performance of self-lubricating polymers. Figure 6 presents the worn surface of P/m-PTFE blends. Figure 6A reveals that an obvious wide and deep abrasion mark was observed, which was likely related to the removal of the polymer substrate arising from the softening of the sample surface. The worn mark became shallower, and the worn surface became smoother with an incremental addition of m-PTFE particles, which was related to the formation of a transfer film on the friction pair and a lubricating film on the surface of the sample block that contributed to improving the tribological performance.

**Figure 7:**
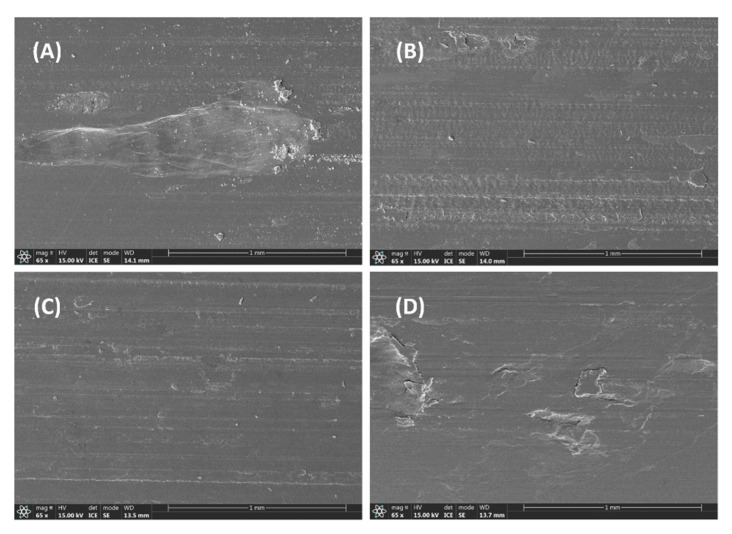
Morphology of the worn surface: (**A**) P/f-PTFE5, (**B**) P/f-PTFE10, (**C**) P/f-PTFE20, and (**D**) P/f-PTFE30.

The worn surface of PPS/f-PTFE blends is presented in Figure 7. Unlike P/m-PTFE5, a clear wear track along with the presence of wear debris is clearly observed in Figure 7A, suggesting that a heavy wear loss likely occurred arising from the accumulation of frictional heat. Furthermore, the worn surface demonstrated more minor tracks along with the presence of fatigue cracks on P/f-PTFE10 and P/f-PTFE20, which was attributed to the plowing or cutting of friction pair due to the existence of some protruding asperities. Moreover, the worn surface of P/f-PTFE30 exhibited obvious worn marks, which was related to the uneven distribution or severe agglomeration of f-PTFE. Under such circumstances, the formation of a uniform and intact transfer film was unfavored, which resulted in poorer friction and wear properties, as displayed in Figure 2C,D.

**Figure 8:**
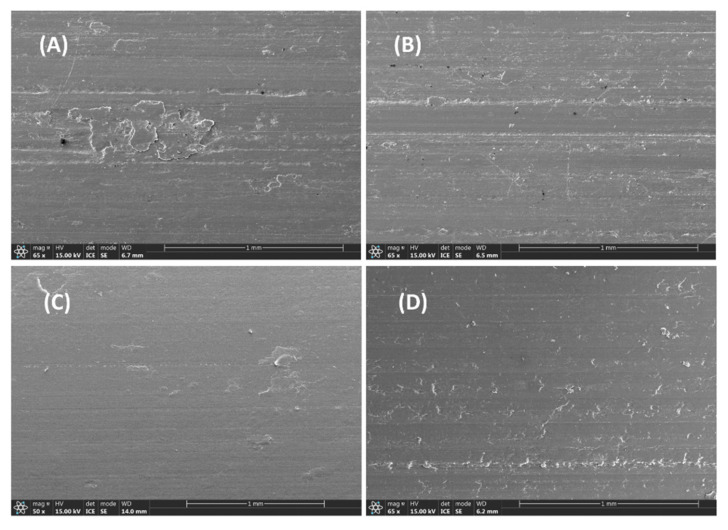
Morphology of the worn surface: (**A**) P/n-PTFE5, (**B**) P/n-PTFE10, (**C**) P/n-PTFE20, (**D**) P/n-PTFE30.

Figure 8 illustrates the morphology of worn surface and wear-mark profiles of PPS/n-PTFE blends. It is evident that some shallow furrows coupling with coil build-up on the worn surface were suppressed with increasing n-PTFE concentrations. Notably, the worn surface of P/n-PTFE20 became smoother and flatter than P/n-PTFE30, and the average wear rate of P/n-PTFE20 was also less than P/n-PTFE30 (see Figure 2F). Under such circumstances, the ununiform distribution of n-PTFE at 30 wt% and the poorer mechanical performance of P/n-PTFE30 in relative to P/n-PTFE20 were considered contributing factors. Additionally, as shown in Figure 8, traces of abrasive debris were present on the worn surface, which is obvious in terms of P/n-PTFE5. Moreover, obvious black-wear debris were observed on the worn surface of P/n-PTFE5 and P/n-PTFE10, as shown in Figure 8A,B. This phenomenon was attributed to the failure of friction sub-surface and related to the accumulation of frictional heat at the sliding interface. As the proportion of n-PTFE increased, the accumulation of frictional heat at the contact surface was reduced, which led to the disappearance of black debris and a reduction in abrasive debris at the edges of the abrasion marks.

## 4. Discussion

After analyzing the mechanical properties of PPS-based blends with different types of PTFE fillers, we concluded that the introduction of PTFE deteriorated the mechanical properties to varying degrees. The P/f-PTFE blends exhibited the most significant reduction in terms of both TS and FS when compared with pure PPS, whereas the P/n-PTFE blends showed a relatively minor decrease among the studied systems. In this scenario, the following factors were considered responsible for such observations: (1) fibrillar PTFE was prone to form physical entanglements within PPS, which resulted in a poor distribution of f-PTFE, thereby significantly weakening the mechanical performance; (2) n-PTFE was treated by irradiation technology, which formed functional groups on the particle surface [39,41] and thereby significantly enhanced the compatibility between n-PTFE and PPS. As a result, the dispersion of n-PTFE was improved which led to a better mechanical performance when compared with that of their f-PTFE- and m-PTFE-containing counterparts.

By comparing the tribological properties among the studied systems, it became evident that incorporating different types of PTFE fillers was favorable for reducing COF and wear loss of PPS. Additionally, the values of average COF decreased consistently with an incremental addition of PTFE fillers, which suggested that incorporating sufficient PTFE particles was favorable for constructing intact transfer film on the surface of friction pair. In this scenario, the presence of continuous transfer film reduced the direct contact between the surface of friction pair and sample block, thereby mitigating the wear resistance. The effectiveness of using PTFE in reducing the tribological properties was related to its ability to form a complete transfer film on the surface of friction pair during sliding process. Moreover, as the weight fraction of PTFE increased, the time required to establish a complete transfer film was significantly reduced, which was related to the easy buildup of PTFE transfer film at higher filler concentrations.

**Figure 9:**
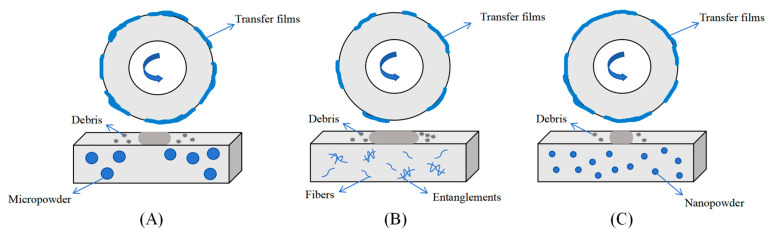
The schematic diagram for tribological measurements: (**A**) m-PTFE; (**B**) f-PTFE; (**C**) n-PTFE.

The schematic diagrams for the tribological measurements regarding different types of PTFE-containing PPS blends were presented in Figure 9. As depicted in Figure 9A, the uneven distribution of m-PTFE was, to some extent, detrimental to the mechanical performance of PPS-blends; however, it favored the formation of transfer film due to the adhesion of PTFE to the sliding metallic counterpart, which reduced the friction and wear performance, especially for the 30 wt% m-PTFE-loaded sample. For example, the average COF and specific wear rate of P/m-PTFE30 reached as low as 0.143 and 1.63 × 10^−6^ mm^3^/Nm, which were 45% and 66% lower than the PA66/PPS/PTFE blend (which had a similar weight fraction of the PTFE fillers) [51]. As observed in the SEM images, f-PTFE formed agglomerates within the PPS substrate, which was disadvantageous for the improvement of mechanical and tribological properties. The n-PTFE had a relatively uniform distribution and it was able to improve the mechanical and tribological properties, as displayed in Figure 9C. As a result, the average COF and specific wear rate of the P/n-PTFE30 blend reached 0.165 and 3.59 × 10^−5^ mm^3^/Nm, respectively, which was 36.5% and 25.2% lower than those of their PA66/PPS/PTFE counterpart (which had a similar weight fraction of the PTFE fillers) [51].

## 5. Conclusions

In this paper, the influence of polytetrafluoroethylene (PTFE) fillers with different morphological geometry on the tribological properties of PPS was systematically investigated. The results revealed that incorporating PTFE impaired the mechanical properties when compared with pure PPS. The tribological performance was greatly improved with an incremental addition of PTFE fillers. Of note, n-PTFE demonstrated optimal modification effect in terms of both mechanical and tribological performance among the studied systems. For example, the average coefficient of friction and specific wear rate of PPS/n-PTFE 30 wt% blends reached 0.165 and 3.59 × 10^−5^ mm^3^/Nm, which were 70.7% and 99.0% lower than pure PPS; meanwhile, their tensile strength and flexural strength reached 50.54 MPa and 84.72 MPa, which demonstrates promising application in industrial sectors. The improved mechanical and tribological performance was attributed to the better compatibility between n-PTFE and PPS, which demonstrates the most optimal integrated modification effect among the studied systems. This work demonstrates the efficacy of using n-PTFE to improve the friction and wear properties of PPS by favoring the formation of a transfer film, showing its potential as a promising solid tribo-modifiers to enhance the tribological performance of polymer composites.

## Figures and Tables

**Table 1:** The formulations of PPS/PTFE composites.

Sample ID	PPS (wt%)	n-PTFE (wt%)	m-PTFE (wt%)	f-PTFE (wt%)
PPS	100	0	0	0
P/n-PTFE5	95	5	0	0
P/m-PTFE5	95	0	5	0
P/f-PTFE5	95	0	0	5
P/n-PTFE10	90	10	0	0
P/m-PTFE10	90	0	10	0
P/f-PTFE10	90	0	0	10
P/n-PTFE20	80	20	0	0
P/m-PTFE20	80	0	20	0
P/f-PTFE20	80	0	0	20
P/n-PTFE30	70	30	0	0
P/m-PTFE30	70	0	30	0
P/f-PTFE30	70	0	0	30

## Data Availability

The original contributions presented in this study are included in the article. Further inquiries can be directed to the corresponding author(s).
